# P01.16. A root extract of Helleborus niger possess cytotoxic properties in neuroblastoma cells

**DOI:** 10.1186/1472-6882-12-S1-P16

**Published:** 2012-06-12

**Authors:** C Delebinski, G Kauczor, P Jesse, K Seeger, G Henze, G Seifert

**Affiliations:** 1 Charité, CVK Berlin, Department of Pediatric Oncology/Hematology, Berlin, Germany

## Purpose

Helleborus niger (Ranunculaceae), commonly known as Christmas rose, is used in anthroposophically extended cancer therapy in the adjuvant treatment of different entities and reduction of chemotherapy-associated side effects. Although Helleborus niger is widely used in anthroposophic medicine, there is a lack of scientific clinical and preclinical data and until now it is applied on an empirical basis. Neuroblastoma is one of the most common extracranial solid tumors of childhood, and more than 50 % of these children initially present with nonresectable primary tumors and disseminated metastasis to distant organ sites, predominantly bone marrow. In this study, we determined for the first time the cytotoxic properties of Helleborus niger Root (HNR) extract for neuroblastoma in vitro.

## Methods

The cytotoxic effect of HNR on the neuroblastoma cell line NXS2 was determined using LDH-assay, mitochondrial membrane potential measurement and Annexin/PI assays. The mechanism of apoptosis was further analyzed by Western blot analysis, caspase inhibitors and mitochondria membrane isolation in more detail.

## Results

We could show that HNR is able to inhibit cell proliferation in a time and concentration dependent manner in NXS2 cells. Furthermore, Annexin/PI and JC-1 assays indicated a dose-dependent induction of apoptosis as the main mechanism of cell death. While western blot analysis revealed a caspase-8 and -9 involvement of apoptosis induction, the incubation with caspase inhibitors did not prevent apoptosis. Moreover, we could detect Ca^2+^ influx from ER into the cytoplasm, which causes Ca^2+^ influx into mitochondria.

## Conclusion

In summary, we demonstrate for the first time a dose- and time-dependent apoptosis induction of HNR in NXS2 cells. Our studies illustrate an involvement of the mitochondrial signaling pathway, whereas the role of caspases remains unclear. To clarify the apoptosis mechanism and the role of calcium and ER stress, further experiments are required.

